# H1N1 Influenza Virus in Patients With Cystic Fibrosis: A Literature Review Examining Both Disease Entities and Their Association in Light of the 2009 Pandemic

**DOI:** 10.7759/cureus.9218

**Published:** 2020-07-16

**Authors:** Dwayne A Wiltshire, Ilmaben S Vahora, Nicholas Tsouklidis, Rajat Kumar, Safeera Khan

**Affiliations:** 1 Internal Medicine, California Institute of Behavioral Neurosciences & Psychology, Fairfield, USA; 2 Medicine, California Institute of Behavioral Neurosciences & Psychology, Fairfield, USA; 3 Health Care Administration, University of Cincinnati Health, Cincinnati, USA; 4 Medicine, Atlantic University School of Medicine, Gros Islet, LCA; 5 Ophthalmology, California Institute of Behavioral Neurosciences & Psychology, Fairfield, USA

**Keywords:** cystic fibrosis, h1n1, influenza

## Abstract

The novel coronavirus (COVID-19) that is challenging the health sector and negatively impacting the global economy takes us back to the 2009 influenza A (H1N1) virus pandemic that brought the world to a standstill. In 2009, H1N1 became a significant health concern for several months. It mainly affected people under the age of 65 hyears who had no prior immunity, including children. Among the high-risk populations were pregnant patients and those with chronic cardiac, pulmonary, or respiratory diseases. These patients were at risk of developing severe pneumonia and respiratory complications. Cystic fibrosis (CF) represents a form of severe chronic lung disease in young adults and is the major fatal hereditary disorder of Caucasians in the United States. An online search of PubMed and Google Scholar was conducted to find relevant literature that explicitly examines patients with CF and H1N1.

## Introduction and background

Cystic fibrosis (CF) is among the significant causes of lung disease researched across the globe. The estimates by the Cystic Fibrosis Foundation Patient Registry indicate that more than 30,000 people, including adults and children in the United States, live with the disease. CF is common among Northern European ancestry and is the most common lethal genetic disease of Caucasians in the United States. On average, the life expectancy of individuals living with the disease is 37.5 years, with many individuals succumbing from severe respiratory failure. CF is an autosomal disorder. A gene mutation coding for the CF transmembrane conductance regulator protein (CFTR) causes defects in the chloride and sodium ions transportation via the epithelial cell membrane. Impaired secretion of these ions, coupled with an increase in absorption of water from the respiratory tract, leads to increased mucous viscosity and defective mucociliary clearance [1]. This illness leads to the impairment of the immune defense and viscid secretions of the respiratory tract. As a result, the patient undergoes chronic microbial infections and constant deterioration in the functioning of the lungs, which is the primary cause of the disease mortality and morbidity. Some of the common bacterial organisms involved in respiratory infections in these patients include *Pseudomonas aeruginosa*, *Haemophilus influenzae*, *Burkholderia cepacia*, and* Staphylococcus aureus* [2]. However, respiratory viruses also play a critical role in disease progression [3]. The patients diagnosed with respiratory infections registered an increased rate of pulmonary exacerbations and CF-related hospitalizations [4]. Patients with CF commonly experience recurrent infections with influenza, parainfluenza, rhinovirus, respiratory syncytial virus, and adenoviruses in a seasonal pattern. The respiratory syncytial virus and influenza have been correlated with a reduction in pulmonary function [3]. Growing evidence indicates that respiratory viruses are associated with the exacerbation of bacterial colonization in CF patients [5]. The influenza A (H1N1) pandemic broke out in 2009 in Mexico and after that spread across the globe. Its incubation period is one to four days. The pandemic primarily affected children and young and middle-aged adults, especially those with chronic respiratory illnesses, such as CF [2]. Today, H1N1 is considered a regular human flu virus with seasonal spikes.

The early initiation of antiviral therapies such as oseltamivir led to a reduction in the disease severity and shortened the duration of viral shedding [6]. The earlier research conducted on the H1N1 pandemic focused on the clinical course and treatment of patients. Some studies have also examined the mutation of this virus and its impact on the severity of influenza [4].

While there have been several studies regarding CF, recent reviews of H1N1 viral infection in this group of patients are limited. However, the emergence of the COVID-19 pandemic has once again brought to the forefront the impact on mortality, morbidity, and economics of pandemic viral illnesses. Accordingly, it is essential to review the impact of H1N1 viral illness on CF patients and use the lessons learned from the past pandemic to deal with the present situation.

## Review

Discussion

The study aimed to examine influenza A (H1N1) in CF patients. An online search via Google Scholar and PubMed was carried out using a combination of the following terms: H1N1 and cystic fibrosis, viral infections and cystic fibrosis. The study then reviewed the articles' abstracts to find the pertinent literature on the topic of interest, thereby including 25 academic articles. Finally, the study was limited to the articles available in English. Table 1 indicates the results of the keyword searches.

**Table 1:** Results of Keyword Searches

Keywords	PubMed	Google Scholar
Cystic Fibrosis	52,875	1,420,000
H1N1	20,802	365,000
Cystic Fibrosis and H1N1	35	6,970
Viral Infections and Cystic Fibrosis	1,280	157,000

**Figure 1:**
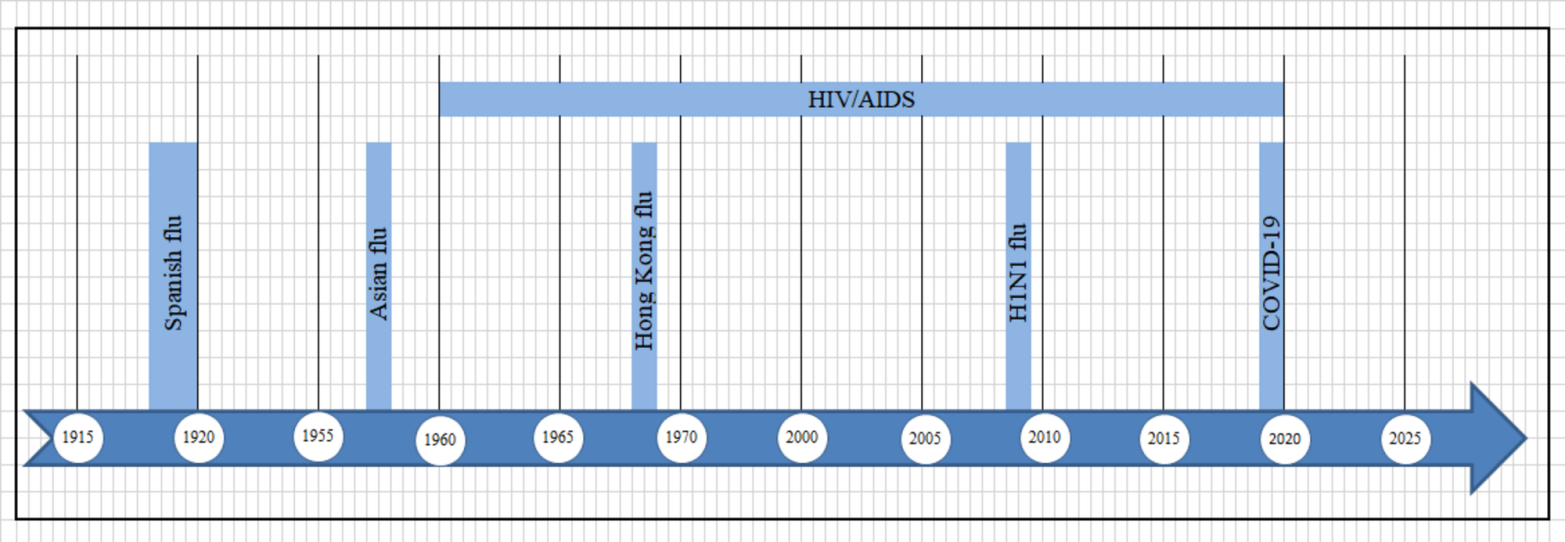
A timeline of crucial pandemics in recent history

The influenza A (H1N1) virus was the cause of the first-ever pandemic in the Northern Hemisphere since 1968 to spread outside the regular flu season. In the contemporary world, it has become a universal human flu virus with variations across the globe. Some groups of people, such as those with asthma, neurological disorders, or chronic immunosuppression, are still vulnerable to severe illness progression. For patients with CF, secondary infection with viral illnesses is a significant element in the management of the disease. Chronic pulmonary disease exacerbations represent a significant percentage of morbidity and mortality. The respiratory viral infections correlate to the decline in the clinical status of CF patients, resulting in a decrease in lung function, nutritional status, and overall health. In older children and adults, the viral illness has the most adverse effects on inhibiting pulmonary function. Surprisingly, our search discovered that the studies representing the impact of the viral pandemic on this group of patients are limited. In the 11 years since its emergence, public health education, adequate antivirals, and vaccine availability have helped reduce this disease's overall effect. Accordingly, this discussion attempts to analyze the available data critically. Figure 1 shows the timeline of crucial pandemics in recent history.

Symptomatology

The signs and symptoms of H1N1 are indistinguishable from other strains of the flu. Approximately 90% of the studies revealed symptoms such as fever, cough, sore throat, and body aches. Gastrointestinal symptoms, such as diarrhea, also occurred. The incubation period ranged from one to four days. Asthma was noted as the most common co-morbidity among hospitalized CF patients with H1N1 [7]. A systematic meta-analysis revealed that fever was the primary symptom in patients with CF presenting with H1N1 [1]. One study found that 94% of the patients had a fever, of which 4.5% had hemoptysis [8]. The production of sputum and fatigue also indicated significant increases with age in these patients. The significant distinction in symptoms between patients with CF and the general population was the increase in sputum production and hemoptysis. There is limited knowledge of hemoptysis mechanisms in patients with CF. 

In another research article aimed at examining the clinical outcomes of H1N1 in adults with CF, no significant difference was found in the symptoms of patients who tested positive via PCR testing vs. those who tested negative [9]. Of note, none of the patients in the H1N1 negative group exhibited sore throat or myalgia.

The symptoms discussed are relatively easy to identify. It is essential to identify warning signs that could lead to a more extended hospital stay and poorer outcomes. Correct symptom pattern identification leads to appropriate testing and early initiation of life-saving interventions.

Hospitalizations

The 1918 Influenza pandemic occurred before the discovery of penicillin by Sir Alexander Fleming. Most deaths during this pandemic were thought to be the result of secondary bacterial pneumonia [10]. The historical pathogens associated with clinical deterioration in patients with CF have been bacterial. Respiratory viruses are now thought to play a significant role in the progression of lung disease in these patients. CF pulmonary exacerbations increase during the winter months and are associated with seasonal influenza [11]. H1N1 can cause a diffuse viral pneumonitis with worsening hypoxemia and progression to shock in the general population. This presentation can be even more debilitating in patients with pre-existing lung disease. A comprehensive study encompassing 4,698 patients from 25 CF centers across the globe indicated that of the 4,698 patients, only 2.3% were infected during the study period, and 50% of those affected needed hospitalization [8]. Accordingly, a systematic meta-analysis conducted to examine the impacts of influenza A (H1N1) on patients with CF revealed that 50%-70% of the CF patients who tested positive for H1N1 required hospitalization [1]. In contrast, this study showed that the general population had a hospitalization rate between 7% and 20%. The significant increase in the hospital admission rate of CF patients must be given consideration.

In the initial stage of the pandemic, the physicians had limited experience in the virus's clinical course. Therefore, they could not establish the criteria for patient hospitalization leading to more people being admitted, including those whose cases could have been managed at home. Therefore, the rate of hospitalization in patients with CF may not correlate with the prevalence of the disease in this population of patients [1]. These studies were conducted using data early within the pandemic. Due to a lack of knowledge of the natural history of the disease and how it progresses in this group of patients, the number of hospital admissions may have been higher than expected. Raised C-reactive protein (CRP) levels and abnormal chest x-ray in patients presenting with influenza-like illness on a background of pulmonary disease should be taken as a sign for possible deterioration and hospital admission [12].

Treatment

Influenza A (H1N1) mostly leads to self-limiting illness. Many patients diagnosed with the disease require bed rest and supportive care with an anticipated recovery of two weeks. However, considerations should be made for the antiviral treatment of all patients who undergo hospitalization. Most importantly, those patients who are at risk or whose immunity is suppressed by pre-existing conditions such as diabetes or cancer should be given priority for antiviral treatment. The two significant antivirals used for the treatment of this respiratory virus are zanamivir taken via inhalation and oseltamivir, which is orally administered [13]. The World Health Organization (WHO) reports that patients with severe illness despite having received oseltamivir treatment should be given licensed antiviral alternatives, such as zanamivir and peramivir. The maximum benefit of these therapies is observed with early initiation, which can shorten the duration of viral shedding [6]. A study conducted to examine the effects of early administration of oseltamivir on viral shedding of the influenza A (H1N1) virus found that patients who received oseltamivir on days 1 to 3 of illness had significantly shorter viral shedding duration in comparison to those treated from day 4 onwards [6].

A meta-analysis trial of CF patients found that 60%-80% of the patients in the studies received oseltamivir antiviral treatment, while antibiotics were administered to around 75%-100% of patients [1]. CF patients have significant pre-existing lung disease and bacterial colonization; hence, aggressive treatment strategies were employed. Bacterial co-infection is linked to significant morbidity and mortality among healthy people [6]. Oseltamivir was well tolerated in patients with CF [14]. However, the H1N1 influenza virus is known to develop oseltamivir resistance via the H275Y oseltamivir-resistance mutation. One case study described an eight-year-old child with CF, who developed resistance after treatment with oseltamivir [15]. Another case study of a 25-year-old woman with CF demonstrated H275Y oseltamivir-resistant H1N1 despite the patient never receiving the drug prior; these findings are suggestive of the community spread of this resistant strain [16]. The resistant strains are, however, less transmissible. The sputum of this 25-year-old patient remained positive for over four months. The impact of such a result on a large scale is not clear. Nevertheless, the treatment of resistant strains is of significant value due to the probability of pulmonary deterioration among CF patients. Research indicates that the new viral strain experiences reassortment with the resistant flu viruses; however, the level of resistance that will appear is not yet established [17].

Prevention

Hygiene Measures

Patients with severe CF are at risk of developing respiratory viral infections, including H1N1 [18]. Consequently, this results in a more extended stay in hospitals, but it also increases the rate of morbidity and mortality among these patients. The healthcare cost in this group of patients has not been well documented. However, the impact that this disease has on CF patients in terms of pulmonary exacerbations calls for adequate implementation of strategies geared towards the prevention of the disease [18]. Infection control in CF centers is a priority [1]. The preventative approaches to reduce H1N1 infections in patients include hygiene measures, vaccination, and chemoprophylaxis with neuraminidase inhibitors such as oseltamivir. Personal hygiene measures include washing hands with soap and clean running water; alternatively, one can use alcohol-based sanitizers. Notably, disinfecting household surfaces with diluted chlorine bleach also reduces the risk of viral transmission. According to expert opinion, handwashing can help prevent viral infection, including the influenza virus. Since influenza spreads through coughs and sneezes, research indicates that droplets containing the virus can stay on surfaces and be transferred to their mouth, eyes, and nose when individuals touch those surfaces with their fingers. Accordingly, anyone with flu symptoms such as cough and fever should stay at home to avoid spreading the virus to the public and get in touch with a doctor for testing. Ideally, social distancing is an excellent strategy to curb the spread and infection of this virus. Social distancing implies that people stay away from one another, and avoid large gatherings such as churches, markets, mosques, and funerals. The public health working closely with other stakeholders has a responsibility to have action plans which call for social distancing actions to be implemented depending on the severity of the outbreak. For hospitalized patients, the WHO recommends that any patient with a suspected infection should be isolated in individual rooms with contact precautions for medical staff. Special care is taken when doing aerosol-generating procedures with the use of N95 masks and negative pressure rooms. Viviani et al. recognized the significance of the implementation of handwashing procedures and contact precautions as strategies for the reduction of the rates of morbidity and mortality among patients with CF [8].

Vaccinations

Another preventative measure against the influenza virus is vaccination. The main aim of vaccination is to prime the immune system of the body so that it responds quickly to the virus. A flu vaccine encompasses either a safe variant of the virus or its proteins to generate immunity. H1N1 vaccinations were specifically developed during the initial outbreak. Trivalent or quadrivalent vaccines are available and protect against several strains of influenza-like illness. Infection with influenza has been shown to worsen pulmonary illness severity resulting in hospitalization [11]. This observation has formed a large part of the rationale for vaccination in these patients. Vaccine refusal is a major public health problem. One French study that included 42 patients with CF found substantial differences in perception of the risks associated with the vaccine compared to being infected with H1N1 [19]. Patients who refused vaccination in this study were not in line with the recommendations of local healthcare providers and were noted to quote multiple contradictory sources. A common problem in the modern world is poor perceptions of vaccinations. The onus is on the healthcare community to combat misinformation and ensure that patients have access to quality evidence-based information to make informed decisions. The patients who accepted the vaccine in the previously mentioned study did so primarily to prevent exacerbations of their disease. A German study looking at barriers to pandemic influenza vaccination found that receiving a seasonal influenza shot was the best predictor of accepting the pandemic viral vaccination [20]. The psychology of habitual behaviors should be furthered examined in newer studies on vaccine compliance. The CF vaccine is well tolerated, and the frequency of side effects is like that of other influenza vaccines [21]. The study mentioned used healthy young patients with functional nutritional status. One limitation noted is that the vaccine efficacy may be lower in patients with more inferior nutritional status or more advanced disease.

Chemoprophylaxis with neuraminidase inhibitors has been recommended by the Center for Disease Control and Prevention (CDC) for individuals exposed to H1N1, once started within 48 hours. Chemoprophylaxis can be considered in unvaccinated patients and in patients in whom vaccines are contraindicated alongside educational strategies [22]. No specific studies on the benefits of chemoprophylaxis in patients with CF were found in the literature. The decision to treat a patient before a confirmatory test with antivirals is based on the prevalence of influenza in the region and the probable benefits of treatment [23].

Prognosis, complications, and mortality

The prognosis of H1N1 in healthy patients is excellent. Most of the patients who died during the pandemic developed severe bacterial pneumonia compounded by other underlying medical illnesses. Pregnant women and patients with rheumatic heart disease had increased mortality rates. One study of patients with CF showed that 48% of patients included were hospitalized for 12.9 days, with 5.4% requiring treatment in the ICU [8]. During the study period, only three people died. These patients already had prior severe lung injury, and another was awaiting transplantation. Another observational study showed that most of their patients only had mild clinical courses and recovered rapidly despite significant symptoms during the initial presentation to a healthcare facility [24]. These studies demonstrate that with appropriate supportive care, H1N1 can be safely managed in relatively healthy patients with CF. A third study done in the United Kingdom showed that 69% of their positive patients required hospital admission; however, none of them required ventilatory support [9]. These studies all demonstrated no significant impact of this illness in this population of patients. The threat of viral infections for progressive respiratory compromise in this group cannot be ignored [25]. Emphasis needs to be placed on preventative strategies and vaccine safety education.

Limitations of this study

The study presents limitations in terms of sample population distinctiveness, generalizability, and reliability. There is no specific distinction between CF populations, whether pediatric or adult. The CF patients are a specialized group of people, and data are often collected retrospectively from CF centers. Data collected during a pandemic are often initially skewed as physician approaches change rapidly with control of disease spread and clinical updates. Also, the study does not consider asymptomatic patients who are infected with H1N1 and represent a carrier state. Notably, there are few meta-analyses of the impact of H1N1 in these patients available.

## Conclusions

Pandemic viral illnesses are challenging for patients with pre-existing lung disease. This article attempts to understand H1N1 in patients with CF. Secondary viral infection presents a concern for acute pulmonary deterioration due to the unique physiology of patients with CF. CF patients infected with H1N1 often have a fever, change in their sputum volume, fatigue, and hemoptysis. As antiviral treatments and effective vaccination became available, the overall impact of this specific influenza-like illness became minimal. Patients with advanced CF and poor nutritional status are at higher risk for complications, prolonged hospitalization, and possible death. Hence, maintaining adequate nutritional state and offering seasonal vaccination for influenzas like illness and H1N1 are critical in this group of patients. This study highlights some of the concerns about pandemic viral illnesses. The preventative strategies used in the previous viral pandemic can be adapted as we approach the novel coronavirus, Covid-19. Future studies should focus on factors preventing vaccine compliance, vaccine effectiveness, and chemoprophylaxis in these patients.

## References

[1] Renk H, Regamey N, Hartl D (2014). Influenza A (H1N1)pdm09 and cystic fibrosis lung disease: a systematic meta-analysis. PLoS One.

[2] Stelzer-Braid S, Johal H, Skilbeck K (2012). Detection of viral and bacterial respiratory pathogens in patients with cystic fibrosis. J Virol Methods.

[3] Kiedrowski MR, Bomberger JM (2018). Viral-bacterial co-infections in the cystic fibrosis respiratory tract. Front Immunol.

[4] Aeffner F, Abdulrahman B, Hickman-Davis JM (2013). Heterozygosity for the F508del mutation in the cystic fibrosis transmembrane conductance regulator anion channel attenuates influenza severity. J Infect Dis.

[5] Billard L, Le Berre R, Pilorgé L, Payan C, Héry-Arnaud G, Vallet S (2017). Viruses in cystic fibrosis patients' airways. Crit Rev Microbiol.

[6] Ling LM, Chow AL, Lye DC (2010). Effects of early oseltamivir therapy on viral shedding in 2009 pandemic influenza A (H1N1) virus infection. Clin Infect Dis.

[7] McKenna JJ, Bramley AM, Skarbinski J, Fry AM, Finelli L, Jain S, 2009 Pandemic Influenza A (H1N1) Virus Hospitalizations Investigation Team (2013). Asthma in patients hospitalized with pandemic influenza A (H1N1) pdm09 virus infection-the United States, 2009. BMC Infect Dis.

[8] Viviani L, Assael BM, Kerem E, ECFS (A) H1N1 Study Group (2011). Impact of the A (H1N1) pandemic influenza (season 2009-2010) on patients with cystic fibrosis. J Cyst Fibros.

[9] Nash EF, Whitmill R, Barker B, Rashid R, Whitehouse JL, Honeybourne D (2011). Clinical outcomes of pandemic (H1N1) 2009 influenza (swine flu) in adults with cystic fibrosis. Thorax.

[10] Morens DM, Taubenberger JK, Fauci AS (2008). Predominant role of bacterial pneumonia as a cause of death in pandemic influenza: implications for pandemic influenza preparedness. J Infect Dis.

[11] Ortiz JR, Neuzil KM, Victor JC, Wald A, Aitken ML, Goss CH (2010). Influenza-associated cystic fibrosis pulmonary exacerbations. Chest.

[12] Nguyen-Van-Tam JS, Openshaw PJ, Hashim A (2010). Risk factors for hospitalization and poor outcome with pandemic A/H1N1 influenza: United Kingdom first wave (May-September 2009). Thorax.

[13] Bearman GM, Shankaran S, Elam K (2010). Treatment of severe cases of pandemic (H1N1) 2009 influenza: review of antivirals and adjuvant therapy. Recent Pat Antiinfect Drug Discov.

[14] Colombo C, Battezzati PM, Lucidi V (2011). Influenza A/H1N1 in patients with cystic fibrosis in Italy: a multicentre cohort study. Thorax.

[15] Esposito S, Molteni CG, Colombo C, Daleno C, Daccò V, Lackenby A, Principi N (2010). Oseltamivir-induced resistant pandemic A/H1N1 influenza virus in a child with cystic fibrosis and Pseudomonas aeruginosa infection. J Clin Virol.

[16] Flight WG, Bright-Thomas R, Mutton K, Webb K, Jones A (2011). Persistent oseltamivir-resistant pandemic influenza A/H1N1 infection in an adult with cystic fibrosis. BMJ Case Rep.

[17] Gangurde H, Gulecha V, Borkar V, Mahajan MS, Khandare RA, Mundada AS (2011). Swine influenza A (H1N1 virus): a pandemic disease. Syst Rev Pharm.

[18] Wang EE, Prober CG, Manson B, Corey M, Levison H (1984). Association of respiratory viral infections with pulmonary deterioration in patients with cystic fibrosis. N Engl J Med.

[19] d'Alessandro E, Hubert D, Launay O, Bassinet L, Lortholary O, Jaffre Y, Sermet-Gaudelus I (2012). Determinants of refusal of A/H1N1 pandemic vaccination in a high-risk population: a qualitative approach. PLoS One.

[20] Böhmer MM, Walter D, Falkenhorst G, Müters S, Krause G, Wichmann O (2012). Barriers to pandemic influenza vaccination and uptake of seasonal influenza vaccine in the post-pandemic season in Germany. BMC Public Health.

[21] Alghisi F, Palma P, Montemitro E, Bernardi S, Pontrelli G, Rossi P, Lucidi V (2011). Immunogenicity and safety profile of the monovalent A/H1N1 MF59-adjuvanted vaccine in patients affected by cystic fibrosis. Thorax.

[22] Uyeki TM, Bernstein HH, Bradley JS (2019). Clinical practice guidelines by the Infectious Diseases Society of America: 2018 update on diagnosis, treatment, chemoprophylaxis, and institutional outbreak management of seasonal influenza. Clin Infect Dis.

[23] Cheng AC-S, Dwyer DE, Kotsimbos TC (2009). ASID/TSANZ guidelines: treatment and prevention of H1N1 influenza 09 (human swine influenza) with antiviral agents. Med J Aust.

[24] France MW, Tai S, Masel PJ, Moore VL, McMahon TL, Ritchie AJ, Bell SC (2010). The month of July: an early experience with pandemic influenza A (H1N1) in adults with cystic fibrosis. BMC Pulm Med.

[25] Zheng S, De BP, Choudhary S (2003). Impaired innate host defense causes susceptibility to respiratory virus infections in cystic fibrosis. Immunity.

